# Effects of seabuckthorn pomace on rumen development, intramuscular fatty acids and antioxidant capacity in weaned lambs

**DOI:** 10.3389/fvets.2025.1560976

**Published:** 2025-04-24

**Authors:** Xiaogao Diao, Xuanzi Zhang, Xiaoyan Hao, Chuntang Mu, Jianxin Zhang

**Affiliations:** ^1^Sanya Institute of China Agricultural University, Sanya, Hainan, China; ^2^College of Animal Science and Veterinary Medicine, Shanxi Agricultural University, Taigu, Shanxi, China

**Keywords:** seabuckthorn pomace, organ weight, rumen development, intramuscular fatty acids, antioxidant capacity

## Abstract

Sea buckthorn pomace (SBP), a by-product derived from sea buckthorn fruit, is rich in nutrients and contains multiple pharmacologically active compounds. Consequently, SBP has the potential to serve as an alternative feed source for ruminants. This study aimed to evaluate the effects of SBP supplementation on organ weight, rumen development, intramuscular fatty acid composition, and antioxidant capacity in weaned lambs. Forty weaned Duper × Small-tailed Han lambs were randomly assigned to one of four dietary treatments in a completely randomized design. The experimental diets included 0% SBP (control), 8% SBP, 16% SBP, and 24% SBP, which were administered over an 80-day period. The results revealed that organ weight increased linearly with SBP supplementation, with the 16% SBP group demonstrating the highest weight gain (*p* < 0.05). Rumen and small intestine deposition exhibited a quadratic response, while omental fat accumulation was significantly greater in the SBP-supplemented groups compared to the control (*p* = 0.01). Additionally, rumen papilla length, width, and keratin layer thickness were positively influenced by SBP supplementation (*p* = 0.01). In rumen fluid, acetate, propionate, total volatile fatty acids, and acetate/propionate ratio showed a linear increase with SBP supplementation, whereas rumen pH displayed an inverse trend (*p* < 0.05). Marbling in longissimus dorsi improved in the SBP group along with enhanced meat quality parameters such as saturated fatty acid (TSFA), total monounsaturated fatty acid (TMUFA), and total polyunsaturated fatty acids (TPUFA), indicating that meat from lambs fed with 16% SBP was more tender and of better quality (*p* < 0.05). Furthermore, SBP also increased the antioxidant capacity of muscle tissue (*p* < 0.05). The above results indicate that adding SBP at a level of 16% in weaned lambs’ diets can enhance organ weight, promote ruminal development, improve meat quality, and provide antioxidant benefits. SBP can be included at up to 24% in weaned lambs’ diets without negative effects.

## 1 Introduction

Sea buckthorn (*Hippophae rhamnoides L*.), a deciduous shrub belonging to *Hippophgae of Elaeagnaceae Juss*, is renowned for its remarkable adaptability to harsh environmental conditions, including drought, salinity, alkalinity, and extreme temperatures (1). Notably, China accounts for approximately 90% of the global cultivation area, encompassing roughly 2.14 million hectares, and produces an annual fruit yield of 500,000 to 600,000 tons, primarily distributed across more than 20 provinces in the northwestern region (2). The berries of sea buckthorn are particularly valued for their rich composition of vitamins, essential amino acids, and flavonoid, which confer a range of bioactive properties, including antioxidant, anti-aging, anti-radiation, and anti-cancer effects. These attributes have led to their extensive utilization in various industries, such as food, cosmetics, nutraceutical, and pharmaceuticals (1, 3). The pomace from sea buckthorn juice extraction, which constitutes approximately 20% of the fresh weight of the berries, consists of skins, seeds, and residual pulp. This byproduct is rich in essential nutrients and antioxidant active components, including flavonoid, organic acids, tannin, and polyphenolic compounds (4, 5). Consequently, SBP holds considerable promise as a valuable unconventional feed resource.

Currently, the livestock industry is confronting a series of global challenges, including rising feed costs, shortages of raw materials, and the misuse of antibiotics. There is an urgent need to identify green, sustainable, and cost-effective feed ingredients. In recent years, Sea Buckthorn Pomace (SBP) has emerged as a promising solution. SBP can be directly utilized as livestock feed after undergoing drying and crushing processes; however, its inclusion rates and effects exhibit variability across different livestock species. Studies indicated that replacing 5% of wheat flour with SBP in the diets of laying hens improved yolk color and increased egg production. Moreover, directly incorporating 5% SBP into broiler diets did not adversely affect the chickens, although it did influence the meat color (6, 7). Dannenberger et al. (8) suggested that SBP-containing diets have a modest impact on fatty acid metabolism in pigs, without significantly affecting hypothalamic–pituitary–adrenal (HPA) axis activity or immune function. Ma et al. (9) reported that the fatty acid composition of the longissimus dorsi muscle in pigs was affected by varying concentrations of SBP and feeding duration, with the highest levels of n-3 fatty acids observed after 8 weeks of feeding a diet containing 12% SBP. In addition, the previous primary study of Hao et al. (10) indicated that the inclusion of 16% SBP in the diets of fattening lambs could enhance the supply of metabolic protein to the gastrointestinal tract, thereby increasing daily weight gain in lambs. However, the effects of SBP on intra-organ growth, rumen development, and meat quality profiles in weaned lambs have not been thoroughly examined. This study hypothesizes that SBP could serve as a potential feed resource in sheep production, enhancing animal performance and productivity. Therefore, this study aims to evaluate the effects of dietary supplementation with varying levels of SBP on intra-organ growth, rumen development, ruminal fermentation characteristics, and meat quality profiles in weaned lambs, providing a theoretical basis for the future utilization of sea buckthorn pomace as an unconventional feed resource in sheep.

## 2 Materials and methods

### 2.1 Animal management and experimental design

Forty healthy 4-month-old Dorper × Small Tail Han ram lambs with an average body weight of 22.20 ± 0.92 kg were selected as experimental subjects. The lambs were randomly divided into four groups based on the proportion of SBP added to their diets: Control group (0% SBP), 8% SBP group (7.80% SBP), 16% SBP group (16.00% SBP), and 24% SBP group (23.5% SBP). Each group consisted of 10 lambs, which were individually housed in pens. The experimental diets were formulated to meet the nutritional requirements for rams weighing 20 kg with a daily gain of 300 g, as recommended by the NRC (2007). SBP was obtained in dry form from a local sea buckthorn juice production facility. The detailed composition and nutritional content of the experimental diets are provided in Table 1. Prior to the experiment, the sheep underwent quarantine, and during the preparatory period, they were dewormed, vaccinated, and the pens were regularly disinfected to ensure cleanliness. The feeding trial lasted 80 days, with feeding conducted at 08:00 and 16:00 daily, allowing for *ad libitum* access to feed and water. All animal handling and experimental procedures were conducted in strict accordance with the guidelines approved by the Animal Care and Ethics Committee of Shanxi Agricultural University (Protocol number: SXAU-[2014]-08).

**Table 1:** Ingredients and chemical composition of experimental diets (%, DM basis).

Item	Treatment			
	Control	8% SBP	16% SBP	24% SBP
Ingredients				
SBP	0.00	7.80	16.00	23.50
Ground corn	28.90	24.20	23.00	14.50
Wheat shorts	5.00	5.00	5.00	5.00
Wheat bran	3.00	3.00	3.00	3.00
Soybean meal	13.40	12.30	12.30	12.30
Oil cake off lax seed	4.70	4.70	4.70	4.70
Oat straw	25.00	27.00	20.00	20.00
Potato plants	15.00	11.00	11.00	12.00
Limestone	0.50	0.50	0.50	0.50
Sodium chloride	0.50	0.50	0.50	0.50
Premix^a^	4.00	4.00	4.00	4.00
Chemical compositions				
CP	13.20	13.20	13.20	13.50
NDF	44.10	41.90	40.50	38.70
ADF	28.40	28.00	26.80	26.90
EE	1.09	2.29	2.60	3.46
NFC	39.60	40.50	41.20	42.00
Ca	0.81	0.75	0.81	0.95
P	0.64	0.63	0.58	0.58
GE,MJ/kg DM	17.70	17.70	17.50	17.80

SBP, sea buckthorn pomace; Control = 0% of DM SBP, 8SBP = 7.8% of DM SBP, 16SBP = 16.0% of DM SBP, 24SBP = 23.5% of DM SBP.

a 5,500 mg/kg of Fe, 51 mg/kg of I, 2,500 mg/kg of Mn, 60,000 mg/kg of S, 11,600 mg/kg of Zn, 1,800,000 IU/kg of vitamin A, 300,100 IU/kg of vitamin D, and 576 IU/kg of vitamin E.

DM, dry matter; CP, crude protein; NDF, neutral detergent fiber; ADF, acid detergent fiber; EE, ether extract; NFC, non-fibrous carbohydrates, NFC, 100-NDF-CP-EE-Ash.

### 2.2 Slaughter and sample collection

Prior to slaughter, the animals were subjected to a 24 h fasting period with water deprivation. On the subsequent day, six sheep from each experimental group were euthanized at the local slaughterhouse. Post-slaughter, various organs were meticulously harvested, weighed, and their weights precisely documented. Approximately 100 mL of rumen fluid was collected, immediately flash-frozen in liquid nitrogen, and subsequently stored at −80°C for the analysis of rumen fermentation parameters. Furthermore, the dorsal sac of the rumen was carefully excised using a scalpel, thoroughly rinsed with physiological saline, and placed in embedding cassettes (2 × 2 cm^2^). These tissue samples were then fixed in 4% paraformaldehyde for subsequent experimental analyses. The longissimus dorsi muscle was harvested following an identical protocol to that used for the rumen tissue collection. A 100 g sample of the longissimus dorsi muscle was collected, flash-frozen in liquid nitrogen, and stored for the determination of fatty acid composition and assessment of antioxidant capacity.

### 2.3 Sample detection and analysis

#### 2.3.1 Observation of rumen and muscle tissue

The rumen dorsal sac, fixed in formaldehyde, was excised and subsequently subjected to dehydration, transparency treatment, infiltration with paraffin, embedding in paraffin, and sectioning. The sections were stained using hematoxylin and eosin (H&E) staining solution, followed by dehydration and mounting. The preparation of muscle tissue sections followed a similar protocol as the rumen tissue. Detailed procedures can be referenced from Natalello et al. (11). The resulting H&E stained sections were observed under a microscope, and the morphological features of both rumen and muscle tissues were recorded. The lengths and widths of papillae and the thickness of the stratum corneum and muscle layers were measured using Image-pro plus 6.0 software (Media Cybernetics, Bethesda, MD, United States).

### 2.4 Rumen fermentation parameters

The pH of rumen fluid was measured immediately using portable pH meter (DDB-303A, Thunder Magnetic Laboratory, Shanghai). Before measurement, the pH meter was calibrated with standard buffer solutions at pH 4.00 and 7.00. The concentration of volatile fatty acids (VFA) in the rumen fluid was determined using gas chromatography (12). The detailed procedure follows: 5 mL of frozen rumen fluid was thawed and centrifuged at 10,000 r/min for 10 min. After centrifugation, 1 mL of the supernatant was transferred to a 4 mL centrifuge tube, to which 0.25 mL of cold 25% metaphosphoric acid was added, followed by 30 min of standing. The mixture was then centrifuged again at 10,000 r/min for 15 min, and 1 μL of the supernatant was analyzed for VFA concentration using a GC-7890B gas chromatography system (Agilent GC7890B, United States). The conditions for determining the content of VFA are as follows: flame ionization detector (FID) and a capillary column (FFAP, 30.00 m × 0.32 mm × 0.50 μm). The heating conditions are: initial temperature of 60°C, increased to 120°C at a rate of 10°C/min, held for 2 min; then increased to 180°C at a rate of 15°C/min, held for 5 min; the vaporization chamber temperature is 250°C. The FID temperature is 250°C; sample volume: 1 μL, carrier gas: high-purity nitrogen (99.99%), pressure: 0.7 MPa; hydrogen pressure: 0.4 MPa, air pressure: 0.4 MPa, capillary column pressure: 0.6 ~ 0.8 MPa, splitless. The concentration of NH_3_-N was determined using the phenol-sodium hypochlorite colourimetric method (13). In this procedure, 3 mL of the thawed rumen fluid was centrifuged at 10,000 r/min for 10 min, and 50 μL of the upper layer was placed in a 10 mL test tube. To this, 3 mL of phenol reagent and sodium hypochlorite reagent were mixed thoroughly and incubated in a water bath at 60°C for 10 min. After cooling, the absorbance was measured at 546 nm using a UV spectrophotometer (721G, Jingke, Shanghai).

### 2.5 Intramuscular fatty acids

The intramuscular fatty acid test was performed according to the procedure of Natalello et al. (11): 100 mg muscle sample was mixed with 2 mL 5% hydrochloric acid methanol, 3 mL chloroform-methanol (1:1) and 100 μL methyl noncognate internal standard in a 15 mL centrifuge tube and subjected mixture to a water bath at 85°C for 1 h. After cooling to room temperature, shocked with 1 mL N-hexane for 2 min and collected 100 μL supernatant after stratification. The supernatant was adjusted to 1 mL with N-hexane and filtered through a membrane with a pore size of 0.45 μm. Finally, the fatty acid content was determined using GC–MS (Trace1310 ISQ, Thermo Fisher Scientific, United States).

### 2.6 Antioxidant capacity

Muscle tissue was homogenized in a 1:1 ratio of tissue weight to physiological saline under ice bath conditions. The homogenate was centrifuged at 12,000 × g for 10 min at 4°C, and the supernatant was collected. The protein content in the liver homogenate was measured using the Bradford assay method (14). Additionally, enzymatic activities of catalase (CAT), total antioxidant capacity (T-AOC), superoxide dismutase (SOD), glutathione peroxidase (GSH-Px), and malondialdehyde (MDA) levels in the muscle were determined using kits from Nanjing Jiancheng Bioengineering Institute.

### 2.7 Statistical analysis

All data was analyzed by One-way Analysis of variance (ANOVA) with SPSS 26.0 software (SPSS For Analytics, version 26; IBM Corporation, Armonk, NY). The linear (L) and quadratic (Q) effects of SBP used Duncan’s multiple comparison test. Means and standard error of the mean (SEM) presented the results, and the significance level is *p* < 0.05.

## 3 Results

### 3.1 Organ weights and fat deposition

Table 2 presented the effects of SBP on organ weights and fat deposition in lambs. The weights of the liver, and kidneys increased linearly with the inclusion of SBP, with the 16% SBP group showing significantly higher weights than the other groups (*p* < 0.05). The spleen, rumen, and small intestine weights exhibited a quadratic effect, with the highest spleen weight observed in the 24% SBP group, in comparison, the weights of the rumen and small intestine in the 16% SBP group were significantly greater than those in all other groups (*p* < 0.05). The weight of the omental fat increased linearly (*p* < 0.05), 24% SBP was the highest, whereas the perirenal fat and tail fat were not significantly influenced by SBP (*p* > 0.05).

**Table 2:** Effects of SBP on the weight of organ and fat in weaned lambs.

Items	Treatment				SEM	*p*-value		
	Control	8% SBP	16% SBP	24% SBP		*T*	*L*	*Q*
The weight of viscera								
Heart, g	145.88^b^	154.29^ab^	164.27^a^	162.19^ab^	2.69	0.05	0.01	0.28
Liver, g	627.97^b^	597.11^b^	696.55^a^	623.49^b^	12.68	0.03	0.02	0.34
Spleen, g	77.08^b^	61.58^b^	71.14^b^	111.01^a^	5.53	0.01	0.01	0.01
Lung, g	370.34	342.65	403.52	380.45	10.09	0.20	0.16	0.91
Kidney, g	92.81^c^	93.87^bc^	108.67^a^	100.41^bc^	1.72	0.01	0.01	0.06
The weight of stomach								
Rumen, g	610.00^c^	705.83^ab^	768.33^a^	667.50^bc^	16.21	0.01	0.04	0.01
Reticulum, g	107.5	104.17	118.33	115.83	2.75	0.10	0.54	0.55
Omasum, g	123.33	148.33	146.67	135.83	4.34	0.15	0.34	0.04
Bomasum, g	185.83	98.33	190.83	224.17	9.18	0.49	0.21	0.58
The weight of intestine								
Small intestine, g	648.33^c^	825.83^b^	967.17^a^	846.67^b^	30.56	0.01	0.01	0.01
Large intestine, g	632.5	633.33	670.00	720.00	15.87	0.17	0.04	0.42
The weight of adipose tissue								
Perirenal fat, g	149.59	187.09	202.14	160.73	9.20	0.16	0.54	0.09
Omental fat, g	230.83^b^	416.67^a^	413.33^a^	423.33^a^	16.26	0.01	0.01	0.03
Tail fat, g	1107.50	999.17	1233.33	1096.28	77.66	0.79	0.79	0.93

SEM, standard error of the mean; *T*, treatment; *L*, linear; *Q*, quadratic. ^a,b,c^ Values within a row with different superscripts differ significantly at *p* < 0.05.

### 3.2 Rumen development

**Figure 1:**
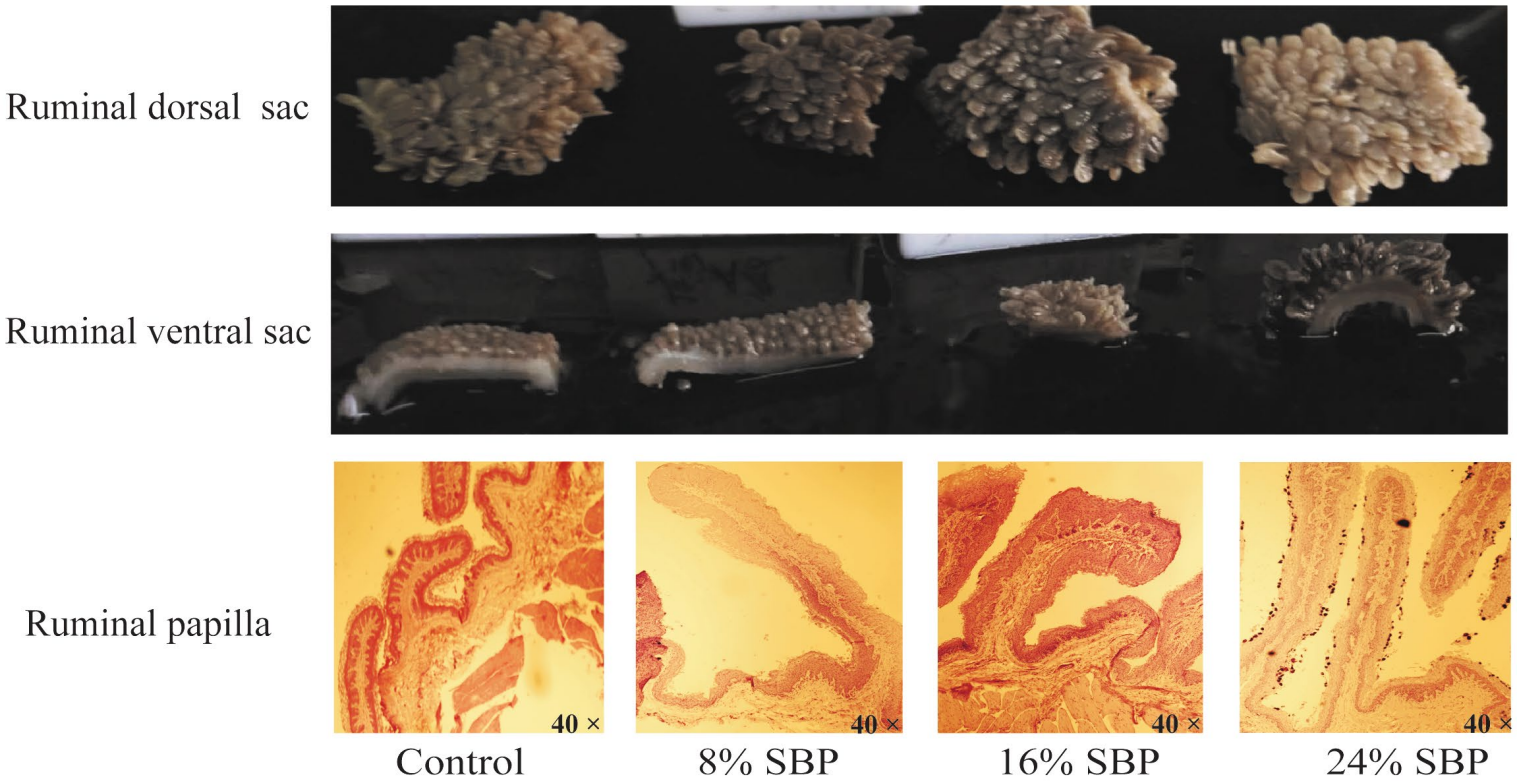
The ruminal dorsal sac and ventral sac morphology in different SBP groups, SBP = sea buckthorn pomace, H&E scale bar = 40X.

Figure 1 illustrated the effects of SBP on the morphological changes of the rumen. Differences in morphology and colouration were observed between the dorsal and ventral sacs of the rumen, with the dorsal sac in the 24% SBP group exhibiting a slightly lighter color than the other groups. In contrast, the ventral sac displayed the deepest colouration. Additionally, histological examination of the HE-stained sections indicated that the length of the ruminal papillae in the SBP groups was greater than that in the control group. Data presented in Table 3 demonstrated that the papillae’s lengths, widths, and keratin levels increased linearly, with both length and width exhibiting a quadratic effect, the 16 and 24% SBP group were superior compared to the other groups (*p* < 0.05), while the muscle layer did not show significant differences (*p* > 0.05).

**Table 3:** Effects of SBP on the rumen morphology in weaned lambs.

Items	Treatment				SEM	*p*-value		
	Control	8% SBP	16% SBP	24% SBP		*T*	*L*	*Q*
Papilla length, μm	2841.21^c^	3262.01^ab^	3421.17^a^	3100.94^bc^	61.98	0.01	0.03	0.01
Papilla width, μm	663.51^c^	756.1^b^	878.94^a^	844.27^a^	20.00	0.01	0.01	0.01
Keratin layer thickness, μm	122.55^c^	128.49^bc^	145.97^a^	138.48^ab^	2.88	0.01	0.01	0.17
Muscle layer thickness, μm	621.1	652.98	695.25	633.6	12.43	0.16	0.45	0.06

SEM, standard error of the mean; *T*, treatment; *L*, linear; *Q*, quadratic. ^a,b,c^ Values within a row with different superscripts differ significantly at *p* < 0.05.

### 3.3 Rumen fermentation parameters

Table 4 presented the rumen fermentation parameters for different groups with varying SBP supplementation levels. The pH of the rumen fluid exhibited a linear decline with the addition of SBP, with the lowest pH recorded in the 24% SBP group (pH = 6.59). The acetate and butyrate concentrations increased linearly, with the highest acetate concentration observed in the 24% SBP group, reaching 40.44 mmol/L. Moreover, the butyrate concentration in the 16 and 24% SBP groups was significantly higher than in the control and 8% SBP groups (*p* < 0.01). The total volatile fatty acid concentration also increased linearly, with the 16 and 24% SBP groups showing significantly elevated levels compared to the control and 8% SBP groups (*p* < 0.01). Additionally, the acetate-to-propionate ratio in the 24% SBP group was significantly higher than in the other three groups (*p* < 0.05). There were no significant changes in NH_3_-N levels (*p* > 0.05).

**Table 4:** Effects of SBP on the rumen fermentation parameters in weaned lambs.

Items	Treatment				SEM	*p*-value		
	Control	8% SBP	16% SBP	24% SBP		*T*	*L*	*Q*
pH	6.89^a^	6.69^ab^	6.71^ab^	6.59^b^	0.05	0.07	0.02	0.7
Acetate, mmol/L	25.95^c^	28.28^b^	35.87^a^	40.44^a^	1.33	<0.01	<0.01	0.36
Propionate, mmol/L	3.29	3.78	4.32	3.72	0.18	0.24	0.25	0.13
Butyrate, mmol/L	4.42^b^	4.24^b^	7.70^a^	7.93^a^	0.53	<0.01	<0.01	0.21
TVFA, mmol/L	37.81^b^	40.45^b^	53.54^a^	54.99^a^	1.89	<0.01	<0.01	0.79
Acetate/propionate	8.31^b^	7.72^b^	8.57^b^	11.02^a^	0.42	0.02	0.01	0.04
NH_3_-N, mg/dL	11.97	12.44	12.12	12.39	0.43	0.98	0.83	0.92

TVFA, total volatile fatty acids; NH_3_-N, ammonia nitrogen; SEM, standard error of the mean; *T*, treatment; *L*, linear; *Q*, quadratic. ^a,b,c^ Values within a row with different superscripts differ significantly at *p* < 0.05.

### 3.4 Intramuscular fatty acids

**Figure 2:**
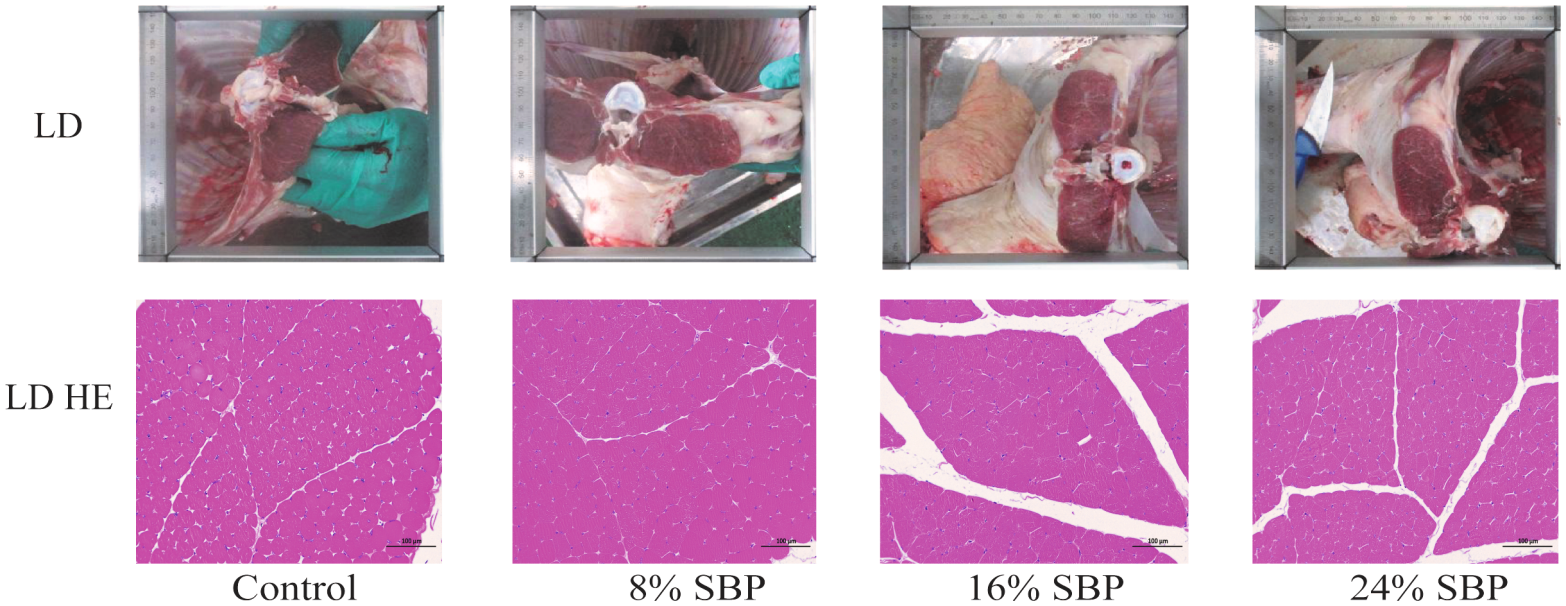
The longissimus dorsi morphology in different SBP groups, LD = longissimus dorsi.

Figure 2 showed the fat distribution in the longissimus dorsi muscle. The images demonstrated that the marbling in the longissimus dorsi muscle of the SBP groups is superior to that of the control group. Additionally, HE staining indicated that the intramuscular fat content was higher in the SBP groups, with the highest levels observed in the 16% SBP group. Table 5 reported the types of intramuscular fat in the longissimus dorsi muscle across different SBP groups and their proportions of total fatty acids (mg/kg). The saturated fatty acid (TSFA), total monounsaturated fatty acid (TMUFA), and total polyunsaturated fatty acids (TPUFA) in the 16% SBP group were significantly higher than those in the other groups (*p* < 0.01). Saturated fatty acids C14:0, C16:0, C17:0, and C18:0 accounted for the highest proportions of total fatty acids, with C14:0, C16:0, C17:0, and C18:0 levels in the 16% SBP group being markedly higher than those in the other groups (*p* < 0.01). Most monounsaturated fatty acids in the 16% SBP group were significantly higher than those in the other groups, except for monounsaturated fatty acid C20:1, which was significantly lower (*p* < 0.01). Similarly, most polyunsaturated fatty acids in the 16% SBP group were significantly higher than in the other groups (*p* < 0.01). There were significant differences in the n-6 and n-3 fatty acids among the SBP addition groups (*p* < 0.01); however, the n-6/n-3 ratio did not differ significantly (*p* > 0.05).

**Table 5:** Effects of SBP on fatty acid profile (g/100 g total FA) of longissimus dorsi muscle in weaned lambs.

Items	Treatment				SEM	*p*-value		
	Control	8% SBP	16% SBP	24% SBP		*T*	*L*	*Q*
C8:0	0.20^b^	0.11^c^	0.29^a^	0.06^c^	0.03	<0.01	0.08	0.04
C10:0	1.46^b^	0.89^b^	3.83^a^	1.04^b^	0.37	<0.01	0.11	<0.01
C12:0	1.04^ab^	0.57^b^	1.51^a^	0.63^b^	0.53	0.09	0.80	0.44
C13:0	0.23^a^	0.04^b^	0.16^a^	0.06^b^	0.03	<0.01	0.008	0.15
C14:0	37.48^b^	21.98^b^	111.48^a^	29.46^b^	11.14	<0.01	0.05	<0.01
C15:0	8.25^b^	2.97^c^	11.32^a^	2.79^c^	1.11	<0.01	0.01	0.02
C16:0	553.62^b^	496.43^b^	894.10^a^	570.44^b^	48.79	0.03	0.13	0.05
C17:0	39.24^a^	17.26^b^	37.41^a^	19.50^ab^	3.93	0.06	0.18	0.74
C18:0	344.11^ab^	255.74^b^	436.03^a^	240.45^b^	25.71	<0.01	0.43	0.16
C20:0	1.36^b^	1.00^b^	2.99^a^	1.02^b^	0.26	<0.01	0.20	0.01
C21:0	1.22^ab^	0.09^c^	0.14^a^	0.09^bc^	0.01	0.01	0.40	0.55
C22:0	0.23^ab^	0.17^c^	0.28^a^	0.21^bc^	0.01	0.01	0.70	0.80
C23:0	0.15	0.12	0.12	0.13	0.01	0.34	0.10	0.24
C24:0	0.10^ab^	0.07^ab^	0.12^a^	0.09^a^	0.01	0.02	0.54	0.98
TSFA	975.98^b^	782.35^b^	1396.87^a^	842.28^b^	85.32	0.01	0.67	0.13
C14:1	0.76^b^	0.40^b^	2.02^a^	0.35^b^	0.23	0.01	0.74	0.03
C15:1	3.35	2.70	3.00	3.39	0.15	0.34	0.77	0.10
C16:1	44.79^b^	40.39^b^	88.16^a^	55.86^b^	6.15	<0.01	0.01	0.04
C17:1	12.67^b^	6.42^c^	21.37^a^	6.86^bc^	1.78	<0.01	0.78	0.05
C18:1n-9c	160.76^b^	138.84^b^	300.56^a^	193.12^b^	19.20	<0.01	0.03	0.10
C18:n-9 t	33.83^b^	34.95^b^	73.47^a^	36.20^b^	4.87	<0.01	0.07	0.03
C20:1	1.22^b^	0.91^b^	2.80^a^	1.15^b^	0.24	<0.01	0.08	0.01
C22.1n-9c	1.13^b^	0.59^b^	0.90^a^	0.67^b^	0.08	0.06	0.10	0.26
C20:1	0.10^a^	0.09^a^	0.05^b^	0.06^b^	0.01	<0.01	0.00	0.13
TMUFA	262.76^b^	221.96^b^	483.58^a^	280.19^b^	34.02	<0.01	0.08	0.05
C20:2	0.35^b^	0.21^b^	0.64^a^	0.32^b^	0.05	<0.01	0.19	0.10
C22:2	0.10	0.07	0.07	0.07	0.01	0.22	0.11	0.27
C18:2n-6c	171.31^b^	124.98^c^	251.23^a^	164.86^b^	13.08	<0.01	0.08	0.14
C18:3n-6	0.87^ab^	0.60^ab^	1.12^a^	0.27^b^	0.13	0.08	0.21	0.20
C20:3n-6	0.04^bc^	0.03^c^	0.14^a^	0.10^ab^	0.02	0.01	0.01	0.61
C20:4n-6	33.38^a^	26.32^b^	35.33^a^	31.65^ab^	1.90	0.02	0.65	0.37
C18:3n-3	36.33^b^	33.04^b^	56.55^a^	43.22^ab^	3.11	0.02	0.05	0.30
C20:3n-3	1.45	1.48	2.07	1.39	0.13	0.15	0.93	0.11
C20:5n-3	13.10^a^	9.26^b^	12.48^a^	9.39^b^	0.57	0.01	0.04	0.64
C22:6n-3	0.65	0.55	0.91	0.81	0.05	0.06	0.06	1.00
TPUFA	257.58^b^	196.56^c^	360.53^a^	251.97^c^	16.89	<0.01	0.06	0.16
n-6	205.60^b^	151.94^c^	287.87^a^	196.89^b^	14.09	<0.01	0.10	0.20
n-3	51.53^b^	44.34^b^	72.00^a^	54.70^b^	3.40	0.01	0.11	0.32
n-6/n-3	4.01	3.45	4.09	3.67	0.16	0.51	0.81	0.85

TSFA, saturated fatty acid; TMUFA, total monounsaturated fatty acid; TPUFA, total polyunsaturated fatty acids; SEM, standard error of the mean; *T*, treatment; *L*, linear; *Q*, quadratic. ^a,b,c^ Values within a row with different superscripts differ significantly at *p* < 0.05.

### 3.5 Antioxidant capacity

Compared to the control group, CAT, TAOC, and GSH-Px exhibited linear changes, while SOD displayed a quadratic effect (Table 6). The activities of CAT, TAOC, and GSH-Px in the 16% SBP and 24% SBP groups were significantly higher than those in the other groups (*p* < 0.05). SOD levels in the 8% SBP and 16% SBP groups were also higher than those in the control and 24% SBP groups (*p* < 0.05). There were no significant differences in MDA levels between the SBP and control groups (*p* > 0.05).

**Table 6:** Effects of SBP on the antioxidative activity in longissimus dorsi muscle in weaned lambs.

Items	Treatment				SEM	*p*-value		
	Control	8% SBP	16% SBP	24% SBP		*T*	*L*	*Q*
CAT, U·mg^−1^prot	3.02^b^	3.13^b^	4.21^a^	3.98^a^	0.16	<0.01	<0.01	0.17
T-AOC, U·mg^−1^prot	1.56^b^	1.95^ab^	2.24^a^	2.29^a^	0.10	0.02	0.01	0.24
SOD, U·mg^−1^prot	14.22^c^	16.91^ab^	17.13^a^	15.37^bc^	0.41	0.01	0.13	0.01
GSH-Px, U·mg^−1^prot	26.87^c^	30.09^bc^	33.20^ab^	37.21^a^	1.23	0.01	0.01	0.82
MDA, nmol·mg^−1^prot	0.62	0.68	0.49	0.6	0.03	0.12	0.27	0.68

CAT, catalase; T-AOC, total antioxidant capacity; SOD, superoxide dismutase; GSH-Px, glutathione peroxidase; MDA, malondialdehyde; SEM, standard error of the mean; *T*, treatment; *L*, linear; *Q*, quadratic. ^a,b,c^ Values within a row with different superscripts differ significantly at *p* < 0.05.

## 4 Discussion

### 4.1 Effects of SBP on organ development and fat deposition in weaned lambs

Seabuckthorn pomace (SBP) is a valuable feed resource containing essential nutrients for livestock and poultry nutrition. And, its organic acid components have been demonstrated to enhance feed palatability and improve feed intake (15). Our previous investigations have established that dietary supplementation with SBP significantly increases dry matter (DM) intake in weaned lambs, accelerates the degradation rates of DM and neutral detergent fiber (NDF), and enhances microbial protein synthesis, consequently promoting body weight gain (10). Organ weight typically accounts for approximately 10% of total body weight, providing insights into the developmental status of livestock (16). The developmental progression and metabolic activities of organs are profoundly influenced by dietary nutrient composition and bioactive compounds. Particularly, plant-derived flavonoid have been extensively documented to exert protective effects and promote organ growth through multiple regulatory pathways (17). Some researches showed that epimedium flavonoid can enhance ovarian antioxidant capacity and reduce ovarian cell apoptosis in laying hens, thereby promoting follicular growth, and supplementation with mulberry leaf flavonoid has been found to exert hepatoprotective effects by reducing blood lipid levels (18, 19). In the present study, the observed increases in heart, liver, rumen, and small intestine weights following SBP supplementation suggest its potential role in promoting organ development in weaned lambs. The effects of seabuckthorn on lipid metabolism appear to be context-dependent. While studies have shown its capacity to reduce fat accumulation through modulation of gut microbiota and regulation of lipid metabolism-related gene expression in high-fat diet models, its application in animal husbandry has demonstrated contrasting effects on fat deposition (9, 20). Previous research found that seabuckthorn pomace can increase thermogenesis by regulating the development of beige adipocytes in young lambs, helping them survive harsh winter conditions (21). Our study’s data show that the fat content around the digestive tract significantly increases with the addition of SBP. Nevertheless, the observed fat deposition cannot be entirely attributed solely to the quantity of SBP added; the increased proportions of EE and NFC in the feed may also contribute to the fat deposition observed.

### 4.2 Effects of SBP on rumen development and fermentation parameters in weaned lambs

The period surrounding weaning in young ruminants is critical for rumen development, which is influenced by dietary composition (22, 23). Previous studies have demonstrated that an optimal ratio of neutral detergent fiber (NDF) to non-fiber carbohydrates (NFC) promotes ruminal papillae growth, whereas an elevated NDF/NFC ratio may exert detrimental effects (24, 25). Although the NDF content in the experimental diets increased with the addition of SBP, the NDF/NFC ratio remained relatively unchanged. Notably, the rumen papillae morphology in the SBP groups were superior to those in the control group. The morphological changes in rumen papillae are primarily stimulated by butyrate in the rumen, as butyrate enhances the mitotic index of rumen papillae epithelial cells and promotes cell proliferation (26, 27). Considering the significant alterations in rumen fermentation parameters, particularly the markedly elevated butyrate levels in both 16 and 24% SBP supplementation groups compared to the control group, the inclusion of SBP substantially modifies the rumen fermentation profile. This modification is characterized by an enhanced butyrate concentration, which consequently stimulates the development of rumen papillae. SBP contains a significant amount of acidic substances, such as malic acid, citric acid, quinic acid, and tartaric acid, which are the primary factors contributing to the reduction in rumen fluid pH (28, 29). The pH of the ruminal fluid in this experiment ranged from 6.59 to 6.89, which is within the normal range. Acetic, propionic, and butyric acids accounted for more than 95% of the total VFAs, serving as the primary energy source for ruminants. The plant flavonoid in animal diets can enhance rumen parameters, thereby providing ruminants with increased energy levels (30). Studies indicates that the addition of sea buckthorn leaf extract to sheep diets significantly boosts the concentration of VFAs (31). Similarly, the supplementation of flavonoid in dairy cow diets elevates the abundance of Firmicutes in the rumen, facilitating the degradation of cellulose and the production of more volatile fatty acids (32). Zhao’s team discovered that sea buckthorn flavonoid can increase the relative abundance of hydrogen-oxidizing bacteria, Ruminococcus, and Rikenellaceae_RC9_gut_group in the rumen bacteria of mid-lactation Holstein cows, leading to enhanced acetate production (33). Furthermore, previous *in vitro* fermentation studies of SBP demonstrated its ability to increase the concentrations of acetate, total volatile fatty acids, and ammonium nitrogen (34). The current feeding trial results align with these findings, showing that SBP can elevate total volatile fatty acids, acetate, and the acetate-to-propionate ratio, although its impact on ammonium nitrogen was not significant. Consequently, SBP may modulate the rumen microbiota, thereby influencing the increase in volatile fatty acid concentrations. The discrepancy in ammonium nitrogen results between this trial and the *in vitro* fermentation study could potentially be attributed to the faster flow rate of rumen fluid *in vivo*.

### 4.3 Effects of seabuckthorn pomace on intramuscular fatty acid in weaned lambs

Meat quality is influenced by multiple factors, including livestock breed, sex, age, feeding regimen, and dietary composition. Adding seabuckthorn leaf powder and flavonoids to the diet can improve poultry carcass and meat quality, such as incorporating 0.4–0.6% seabuckthorn fruit flavonoids in broiler diets can affect growth performance and fat deposition. Now, seabuckthorn pomace has been found to enhance muscle quality and alter the distribution of muscle fiber size in lambs (35, 36). In the present study, histological examination of the longissimus dorsi muscle through H&E staining revealed a significant increase in intramuscular fat content, which is consistent with previous findings from our research group (37). Intramuscular fatty acids are one of the key factors influencing lamb flavour, which is affected by feeding practices and diet composition (38, 39). Under intensive feeding conditions, meat sheep can accelerate fat deposition and increase the levels of mutton flavour compounds. However, incorporating herbal plants such as rosemary, garlic, and ramson into the diet can significantly reduce the amounts of these flavour compounds (40, 41). For instance, alfalfa supplementation has been demonstrated to enhance the content of conjugated linoleic acid isomers, omega-3 fatty acids, and alpha-tocopherol in lamb meat (42). Similarly, ramson extract has been found to significantly increase the concentrations of total saturated and monounsaturated fatty acids in lamb (43). Current research indicates that dietary supplementation with specific plant materials or extracts can modulate the composition and concentration of intramuscular fatty acids while preserving flavour compounds in lamb meat, thereby influencing post-mortem flavour characteristics (44). In this investigation, both the 8 and 24% SBP groups exhibited the lowest total saturated fatty acid content, with the levels of C8:0, C10:0, and C12:0—compounds associated with the mutton flavour—also being lower than those in control and 16% SBP groups, suggesting that SBP may effectively reduce mutton flavour. However, the 16% SBP group demonstrated significantly higher levels of saturated fatty acids, monounsaturated fatty acids, and polyunsaturated fatty acids than the other groups, possibly due to this group’s higher intramuscular fat content. Furthermore, seabuckthorn contains various saturated and unsaturated fatty acids, and its flavonoids may enhance the ratio of unsaturated to saturated fatty acids in poultry muscles (9, 45), all of which are factors influencing the results of this study. Based on the analysis of intramuscular fat content and fatty acid composition in the longissimus dorsi muscle, the supplementation of SBP demonstrates significant benefits in promoting the development of “marbled mutton” characteristics. These findings suggest that SBP can serve as a valuable dietary supplement for modulating meat quality parameters in weaned lambs.

### 4.4 Effects of seabuckthorn pomace on the antioxidant capacity of muscles in weaned lambs

Dietary supplementation with antioxidant compounds has been demonstrated to enhance the antioxidant capacity of lamb meat, thereby improving meat quality parameters and extending shelf life. The plant extracts containing polyphenols and flavonoids are the primary choice for dietary antioxidants in animal feed, second only to vitamin E (46, 47). Chikwanha et al. (48) found that feeding grape pomace can enhance the antioxidant capacity of lamb meat, delaying lipid and protein oxidation, thereby extending the meat’s shelf life. Adding seabuckthorn leaves and chromium to the diet significantly reduced the degree of lipid oxidation in chickens during frozen storage. Furthermore, using seabuckthorn alone in chicks can alleviate the immunosuppression caused by T-2 toxin (49, 50). In this study, the incorporation of 16 and 24% SBP significantly enhanced the antioxidant capacity of muscle, positively impacting the quality of lamb meat. However, further investigation is required to elucidate the underlying mechanisms through which SBP improves lamb meat quality and to determine its efficacy in shelf life extension.

## 5 Conclusion

In conclusion, this study demonstrates that SBP does not exert any adverse effects on weaned lambs. The addition of SBP alters the rumen fermentation pattern, enhances the production of volatile fatty acids, and promotes rumen development. Furthermore, SBP can increase intramuscular fat deposition and antioxidant capacity, thereby improving meat quality. Based on comprehensive analysis of various experimental indicators, this study suggests that the optimal dietary inclusion level of SBP should range from 16 to 24%.

## Data Availability

The original contributions presented in the study are included in the article/supplementary material, further inquiries can be directed to the corresponding author.
